# Apical Hypertrophic Cardiomyopathy With Endomyocardial Calcification: A Multimodality Imaging-Based Case Report

**DOI:** 10.7759/cureus.86822

**Published:** 2025-06-26

**Authors:** Sho Tanabe, Chisato Takamura, Masahiro Terashima

**Affiliations:** 1 Cardiology, Cardiovascular Imaging Clinic Iidabashi, Tokyo, JPN

**Keywords:** apical hcm, cardiovascular magnetic resonance imaging (cmr), endomyocardial calcification, myocardial perfusion reserve, myocardial strain

## Abstract

Apical hypertrophic cardiomyopathy (ApHCM) with endomyocardial calcification has been reported in only a small number of cases, making its imaging features less well established. We describe a 53-year-old woman with no significant medical history who was referred for further evaluation following abnormal electrocardiographic findings identified during a routine medical check-up. Multimodality imaging, including transthoracic echocardiography, coronary computed tomography angiography, and cardiac magnetic resonance imaging (CMR), demonstrated apical myocardial hypertrophy and endomyocardial calcification, establishing the diagnosis of ApHCM with calcific involvement. The pathophysiological mechanism underlying myocardial calcification in ApHCM remains poorly defined. In this case, adenosine stress perfusion CMR and myocardial strain analysis were performed for further characterization. These investigations revealed a circumferential stress-induced perfusion defect predominantly involving the apical myocardium, along with markedly reduced apical global circumferential strain (GCS), suggesting a potential association with chronic subendocardial ischemia; however, this remains a hypothesis based on a single case and requires further validation. The use of adenosine stress perfusion CMR in this case was intended to evaluate for coexisting ischemia, given the presence of calcification and impaired apical strain. Clinically, identifying calcification rather than thrombus is critical, as it may prevent unnecessary anticoagulation and guide appropriate follow-up strategies. This case underscores the importance of a multimodality imaging approach in assessing both structural and functional alterations in atypical phenotypes of hypertrophic cardiomyopathy.

## Introduction

Apical hypertrophic cardiomyopathy (ApHCM) is a morphological subtype of hypertrophic cardiomyopathy (HCM), characterized by predominant thickening of the left ventricular apex. While ApHCM is relatively uncommon in Western populations, it is more frequently observed in East Asian countries, particularly in Japan, where it accounts for up to 15% of all HCM cases, compared to approximately 3% in the United States [1]. Many patients are asymptomatic, and the condition is often diagnosed incidentally based on abnormal electrocardiographic findings identified during routine health evaluations.

Transthoracic echocardiography (TTE) is widely used as an initial diagnostic tool for suspected cardiomyopathy. However, visualization of the apical region can be technically limited, particularly in early or borderline cases, which may lead to underdiagnosis [2]. In some patients, ApHCM may be complicated by apical aneurysm formation or intracavitary thrombus, both of which may require anticoagulation or surgical intervention [3].

ApHCM accompanied by endomyocardial calcification has been described in only a limited number of reports, and its clinical and imaging features remain poorly understood [4-6]. Owing to the scarcity of documented cases, reliable data on the incidence or prevalence of this condition are currently unavailable. Although endomyocardial calcification is generally considered less thrombogenic than intracavitary thrombus, it may impair myocardial compliance and contractility, contributing to left ventricular dysfunction. The underlying pathogenesis remains unclear. One hypothesis is that chronic subendocardial ischemia caused by apical cavity obliteration leads to progressive myocardial fibrosis and subsequent dystrophic calcification [7]. This proposed mechanism may be supported by stress perfusion CMR findings, which allow for noninvasive detection of regional perfusion defects. Additional contributing factors may include metabolic disturbances (such as diabetes), chronic renal insufficiency, or prior inflammatory myocardial injury [8]. However, in the present case, the patient had no history of metabolic or inflammatory disease, and we considered the ApHCM itself to be the most plausible contributor to the calcification.

Accurate differentiation between endomyocardial calcification and thrombus is essential for proper diagnosis and therapeutic decision-making. Cardiac magnetic resonance imaging (CMR) and coronary computed tomography angiography (CCTA) provide superior tissue characterization for this purpose. Moreover, CMR uniquely allows assessment of myocardial perfusion and strain, enabling a comprehensive structural and functional evaluation in patients with atypical HCM phenotypes, such as ApHCM. In this context, it is also important to consider other potential mimickers such as apical tumors or dense fibrosis, although these are typically distinguishable by imaging characteristics. The application of multimodality imaging, especially CMR-based perfusion and strain analysis, may enhance understanding of disease mechanisms and support clinical management in uncommon presentations such as this.

## Case presentation

**Figure 1:**
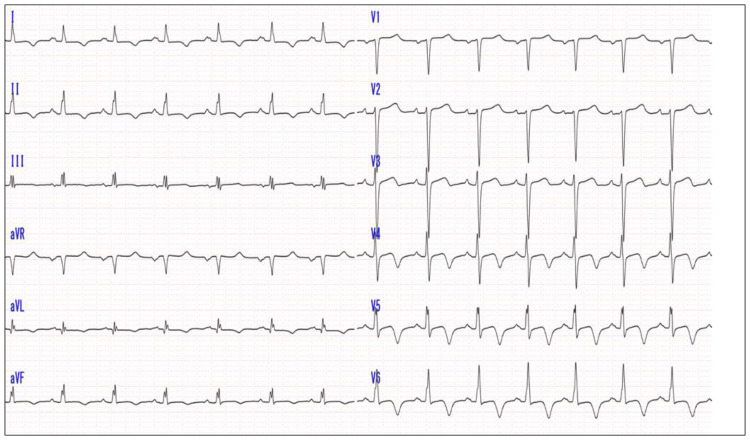
A 12-lead electrocardiogram showing T-wave inversion in leads I, II, III, aVF, and V2-V6.

The patient is a 53-year-old woman who was referred for further detailed evaluation after an abnormal electrocardiogram was noted during her annual medical check-up. She had no relevant past medical history or family history of cardiovascular disease. The electrocardiogram demonstrated sinus rhythm and T-wave inversion in leads I, II, III, aVF, and V2 through V6 (Figure 1).

**Figure 2:**
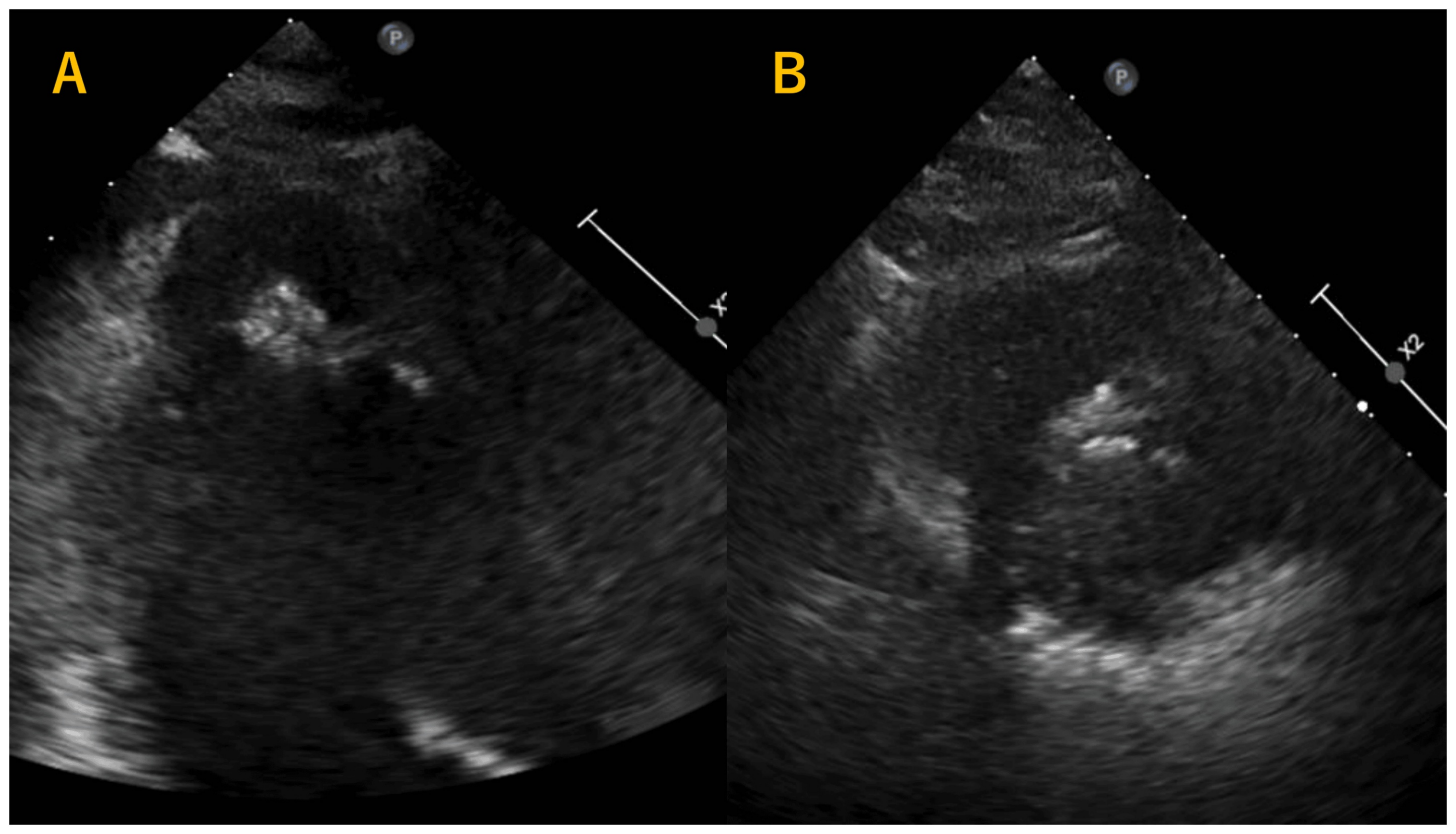
Transthoracic echocardiography. (A) Three-chamber view. (B) Short-axis view. Both panels show apical endomyocardial calcification.

TTE revealed preserved left ventricular systolic function (ejection fraction: 72%), hypertrophy of the apical myocardium, and evidence of apical endomyocardial calcification (Figure 2). The apical lesion appeared immobile and highly echogenic on TTE, favoring a diagnosis of calcification rather than thrombus.

**Figure 3:**
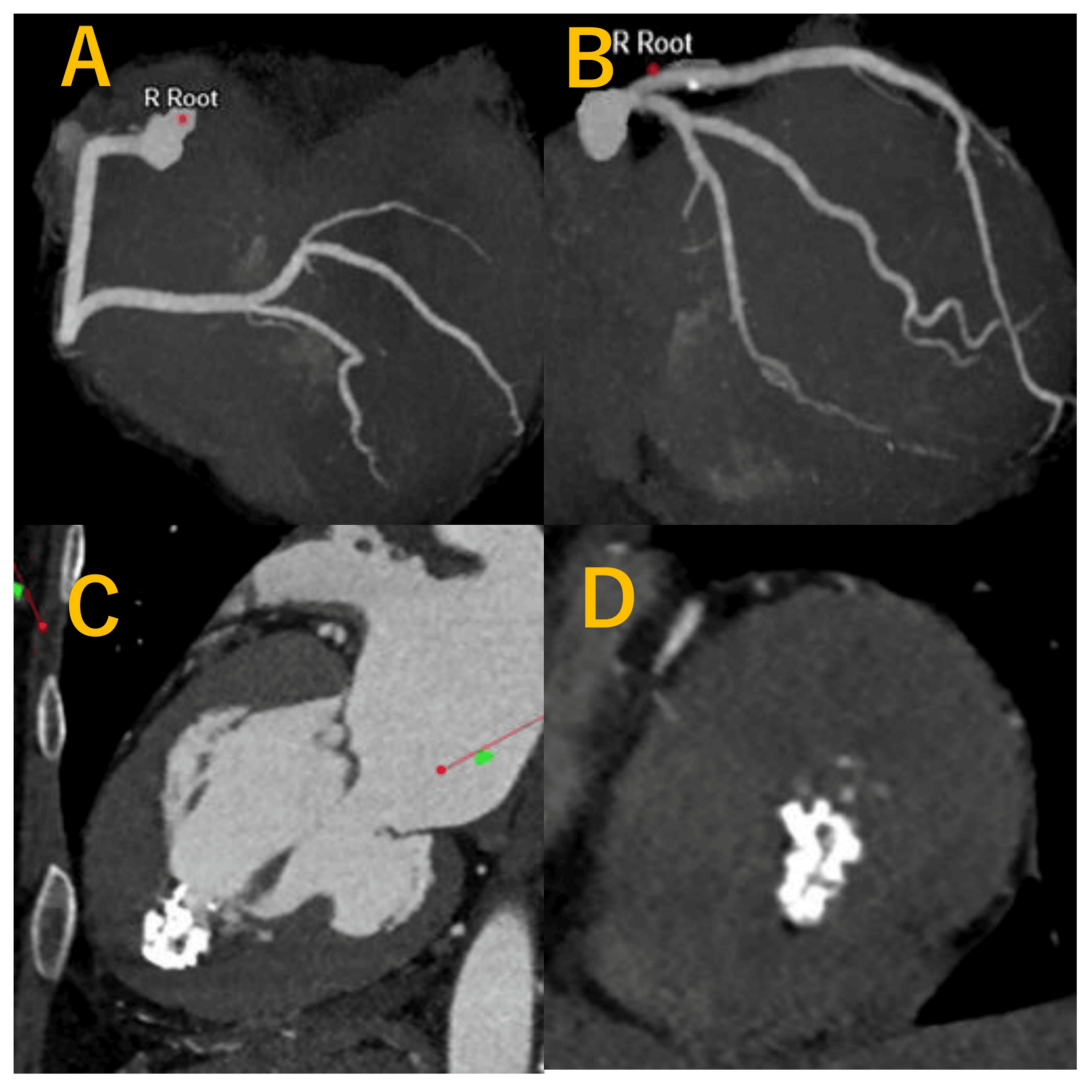
Coronary computed tomography angiography (CCTA) showing no significant stenosis in the right (A) and left (B) coronary arteries. Apical hypertrophy with endomyocardial calcification is evident in the left ventricle (C, D), with no evidence of thrombus.

Subsequently, CCTA was performed to rule out ischemic heart disease. CCTA revealed no significant coronary artery stenosis (Figures 3A-3B), and identified a hyperdense, sharply marginated lesion at the apex (Figures 3C-3D), morphologically distinct from typical left ventricular thrombus. These findings from TTE and CCTA supported the diagnosis of endomyocardial calcification.

Finally, CMR was performed. Cine CMR demonstrated a preserved left ventricular ejection fraction of 63%, consistent with maintained global systolic function. 

**Figure 4:**
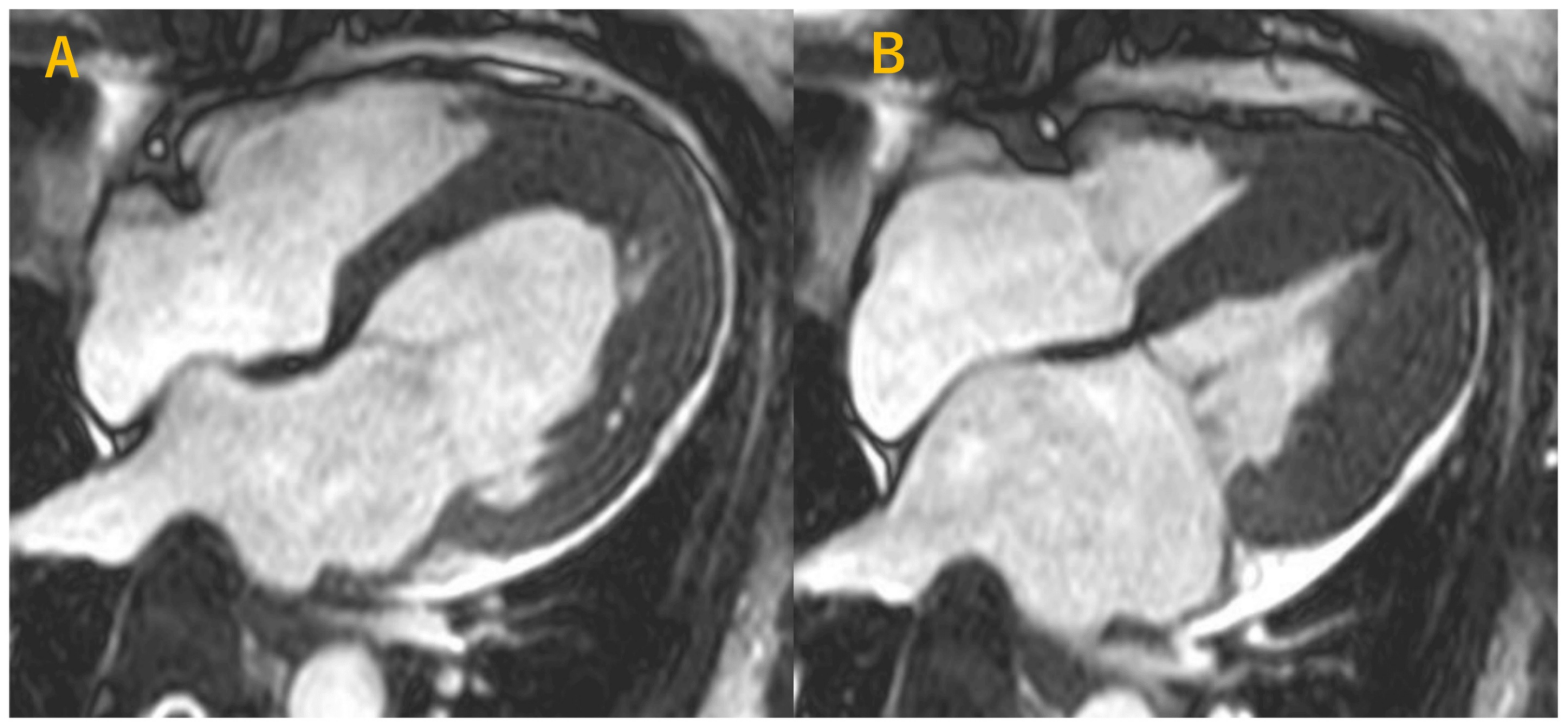
Cine cardiac magnetic resonance imaging (four-chamber view). (A) End-diastolic frame. (B) End-systolic frame. Both panels show a spade-shaped configuration and marked apical hypertrophy of the left ventricle.

However, a distinct spade-shaped configuration of the left ventricle was observed (Figure 4).

**Figure 5:**
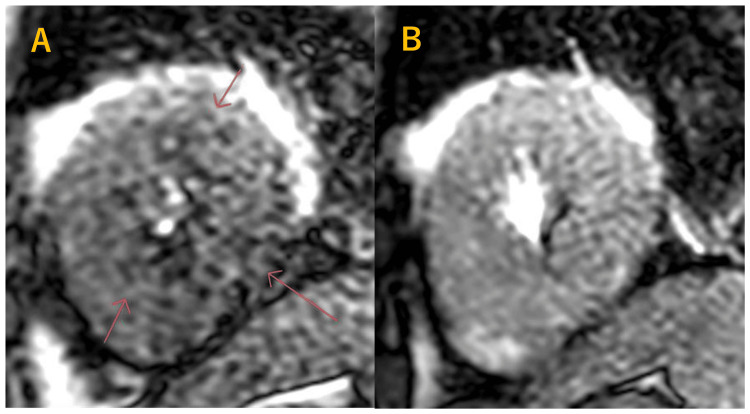
Stress perfusion cardiac magnetic resonance imaging. (A) Stress phase showing a circumferential stress-induced perfusion defect predominantly in the apical region. (B) Rest phase showing no corresponding perfusion defect.

T2-weighted black-blood imaging (T2WBB) demonstrated high signal intensity in the apical region. This finding was interpreted as myocardial edema, a common feature of ApHCM, and did not contribute directly to the diagnosis of calcification. In this case, we also did stress CMR myocardial perfusion imaging under adenosine-induced stress. Stress perfusion imaging revealed a circumferential stress-induced perfusion defect predominantly affecting the apical region (Figure 5).

Late gadolinium enhancement (LGE) demonstrated subepicardial-dominant enhancement at the apex, with areas of transmural enhancement also observed.

**Figure 6:**
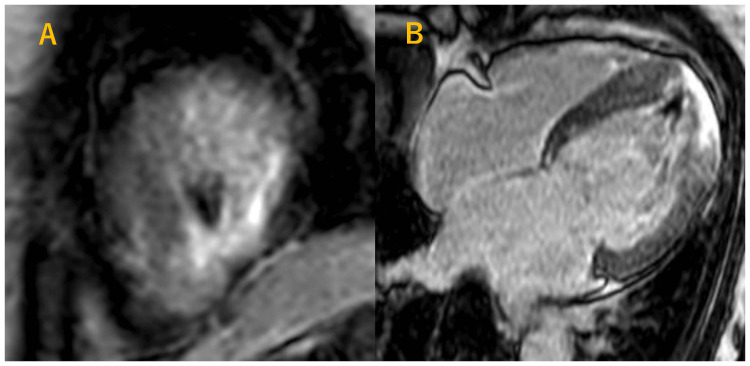
Late gadolinium enhancement images. (A) Short-axis view. (B) Four-chamber view. Both panels show apical subepicardial and transmural enhancement, with a low-signal endocardial area suggestive of thrombus or calcification.

A low-signal intensity area was noted on the endocardial side, raising suspicion for thrombus or calcification (Figure 6).

**Figure 7:**
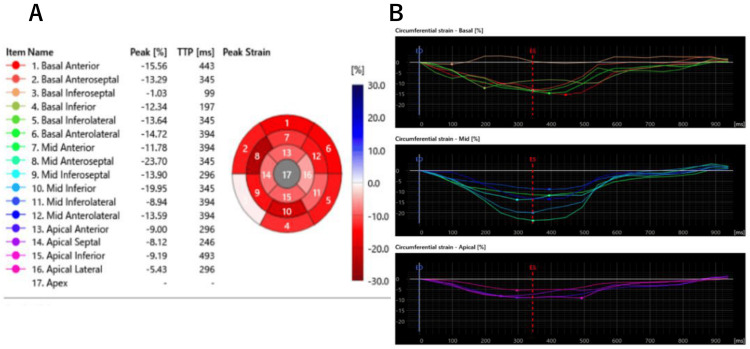
Global circumferential strain (GCS) analysis based on cardiac magnetic resonance imaging. (A) Bull’s eye plot demonstrating reduced strain in the apical segments. Strain values are color-coded from dark red (more negative, indicating preserved contraction) to light red and white (less negative, indicating impaired contraction). (B) Corresponding segmental strain curves for the basal, mid, and apical levels (top to bottom), confirming predominant apical dysfunction.

Subsequently, additional strain analysis of the left ventricular myocardium was performed using IntelliSpace Portal (ISP) (Philips Medical Systems, Best, Netherlands). The results demonstrated a marked reduction in global longitudinal strain (GLS), global radial strain (GRS), and global circumferential strain (GCS), predominantly in the apical region (Figure 7).

In particular, the reduction in GCS at the apical segment was striking.

The apical GCS was -7.94%, which is markedly lower than the typical value of approximately -20% in healthy individuals, and also significantly reduced compared to previously reported values in ApHCM. This finding suggests substantial regional dysfunction, possibly associated with calcification.

Based on the comprehensive assessment of findings from TTE, CCTA, and CMR, a diagnosis of ApHCM with apical myocardial calcification was made.

Although CMR alone could not completely rule out the presence of thrombus in the apex, the combined interpretation of CCTA and TTE findings supported the diagnosis of myocardial calcification in the left ventricular apex.

Because no definite thrombus was identified, anticoagulation therapy was withheld. The patient was enrolled in continuous arrhythmia monitoring and scheduled for follow-up TTE and CMR every six months.

## Discussion

We report a case of a 53-year-old woman with ApHCM accompanied by endomyocardial calcification. This case presents an interesting set of imaging findings observed in a patient with ApHCM and highlights three clinically relevant aspects.

First, differentiating endomyocardial calcification from thrombus in the left ventricle remains a diagnostic challenge. Although TTE is widely used, its ability to visualize the apical region is limited. Previous studies have reported that TTE detects only approximately 23% of left ventricular thrombi [9]. CMR offers superior apical visualization and is highly sensitive for thrombus detection; however, dense calcification and thrombus can show overlapping signal characteristics, making distinction difficult. In the present case, the use of multiple imaging modalities, including TTE, CCTA, and CMR, was helpful in supporting the diagnosis of endomyocardial calcification rather than thrombus [10].

Second, stress perfusion cardiac MRI revealed a circumferential perfusion defect predominantly involving the apical myocardium, indicating significant subendocardial ischemia. Hughes et al. previously demonstrated that myocardial perfusion reserve is reduced across all phenotypes of hypertrophic cardiomyopathy, with the most pronounced reduction seen in overt ApHCM and asymmetric septal hypertrophy, particularly in the subendocardium [11]. In that study, the average apical perfusion reserve in overt ApHCM was 1.61. In contrast, our patient exhibited a lower value of 1.28, which falls below the commonly proposed threshold of 1.5 for impaired myocardial perfusion and may suggest the presence of microvascular dysfunction. This value was calculated as the ratio of myocardial blood flow during stress to that at rest, consistent with the methodology employed in the referenced study. Although absolute myocardial blood flow values may vary depending on the imaging system and post-processing software, the use of a ratio mitigates inter-system variability and allows for a reasonably reliable comparison. This case highlights the potential utility of stress perfusion cardiac MRI in characterizing myocardial abnormalities in ApHCM with endomyocardial calcification. The observed findings may support a hypothesis-generating association between chronic subendocardial ischemia and dystrophic myocardial calcification, although a causal relationship cannot be established from a single case.

Third, myocardial strain analysis using cardiac MRI-based techniques demonstrated a marked reduction in apical GCS. The normal apical GCS in healthy individuals has been reported to be approximately -19.6% [12]. Previous studies have shown that GLS, GRS, and GCS are significantly reduced in patients with ApHCM compared to healthy individuals, and these parameters are known to correlate with disease severity [13]. Wang et al. reported that the mean apical GCS in typical ApHCM without calcification was approximately -15.9 ± 5.3% [14]. In contrast, our patient exhibited a value of -7.94%, indicating more severe regional dysfunction. Strain in the mid-ventricular and basal segments was mildly reduced, consistent with typical patterns observed in ApHCM, but less pronounced than in the apical region. This substantial reduction in apical strain may reflect localized mechanical impairment associated with myocardial calcification, potentially secondary to chronic ischemia and fibrosis. While additional studies involving larger case series are necessary to confirm this observation, our findings suggest that severely impaired apical strain may serve as a potential imaging marker for subclinical myocardial damage or early calcific remodeling in ApHCM.

## Conclusions

This case describes an uncommon combination of ApHCM with endomyocardial calcification, which has been infrequently reported in the literature. In clinical settings, differentiating calcification from thrombus using CMR alone can be challenging, as both may appear as low-signal-intensity regions. In this patient, multimodality imaging, including TTE and CCTA, was helpful for diagnostic clarification and may have contributed to the decision to avoid anticoagulation and pursue conservative management. This report primarily aims to present an interesting set of imaging findings observed in a case of ApHCM using multiple imaging modalities. Although the mechanism of myocardial calcification in ApHCM remains unclear, the observed findings suggest a hypothesis-generating association with chronic subendocardial ischemia and regional mechanical dysfunction. This report is limited by the absence of histopathological confirmation and the inherent constraints of a single case. Further studies, including multicenter registries and longitudinal imaging-outcome analyses, are warranted to better understand the nature and clinical implications of this phenomenon.
